# Supplementary material for: Transcriptomic and Chromatin Landscape Analysis Reveals That Involvement of Pituitary Level Transcription Factors Modulate Incubation Behaviors of Magang Geese
Source: Genes (Basel). 2023 Mar 28;14(4):815. doi: 10.3390/genes14040815 (PMC10137868; doi:10.3390/genes14040815)
Supplement: Supplementary file 1 [file genes-14-00815-s001.zip › genes-2299825-supplementary/Supplemental Tables and Figures/SupplementalFigure.pdf]

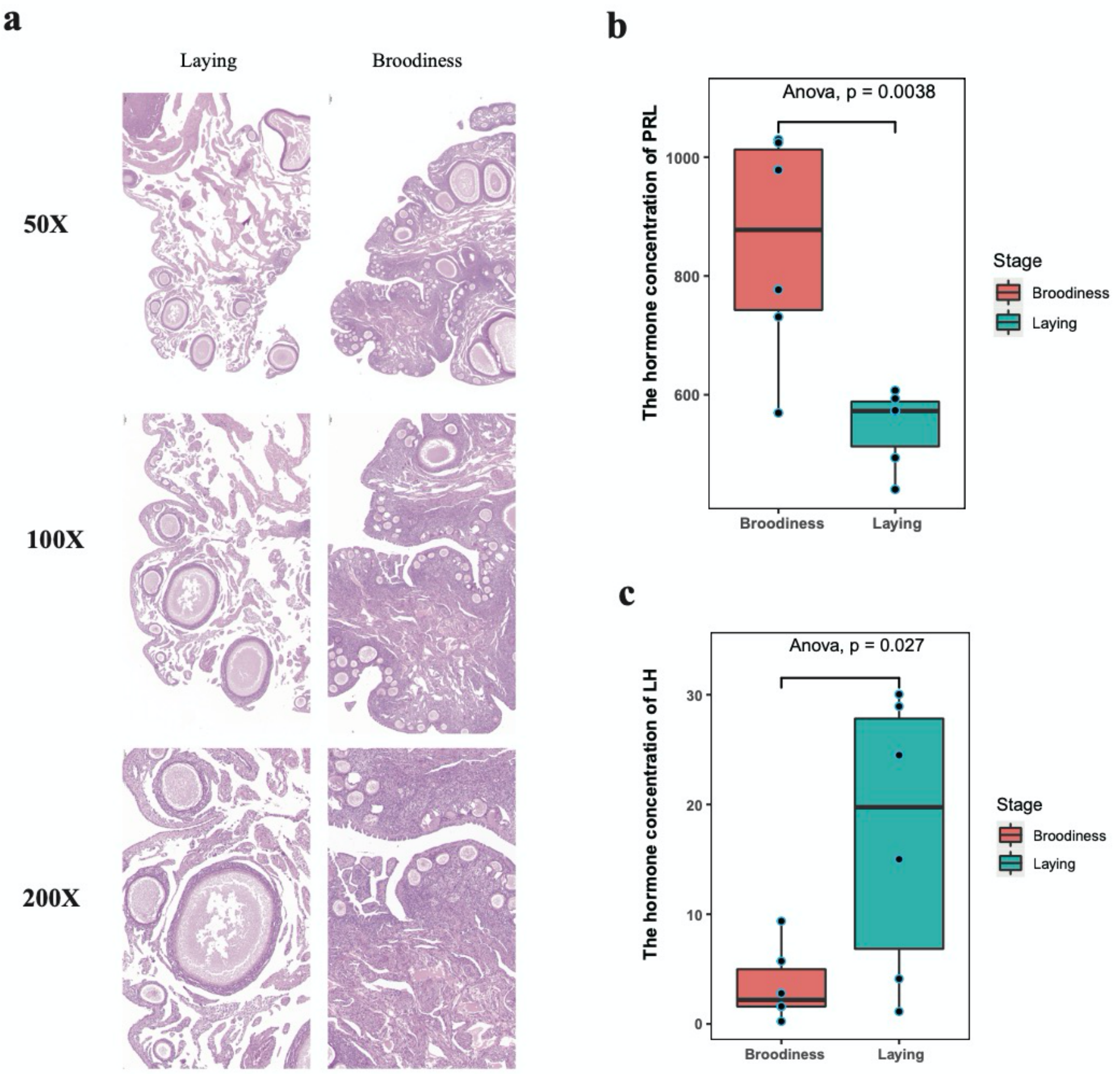

Figure S1. Ovarian histology and hormone concentration in Magang geese. (a) Hematoxy- lin-staining of the geese ovarian tissue in both laying and brooding stages. (b) The serum concentration of PRL between the laying and the brooding stages. (c) Changes of LH con- centration in serum between the laying and brooding stages.

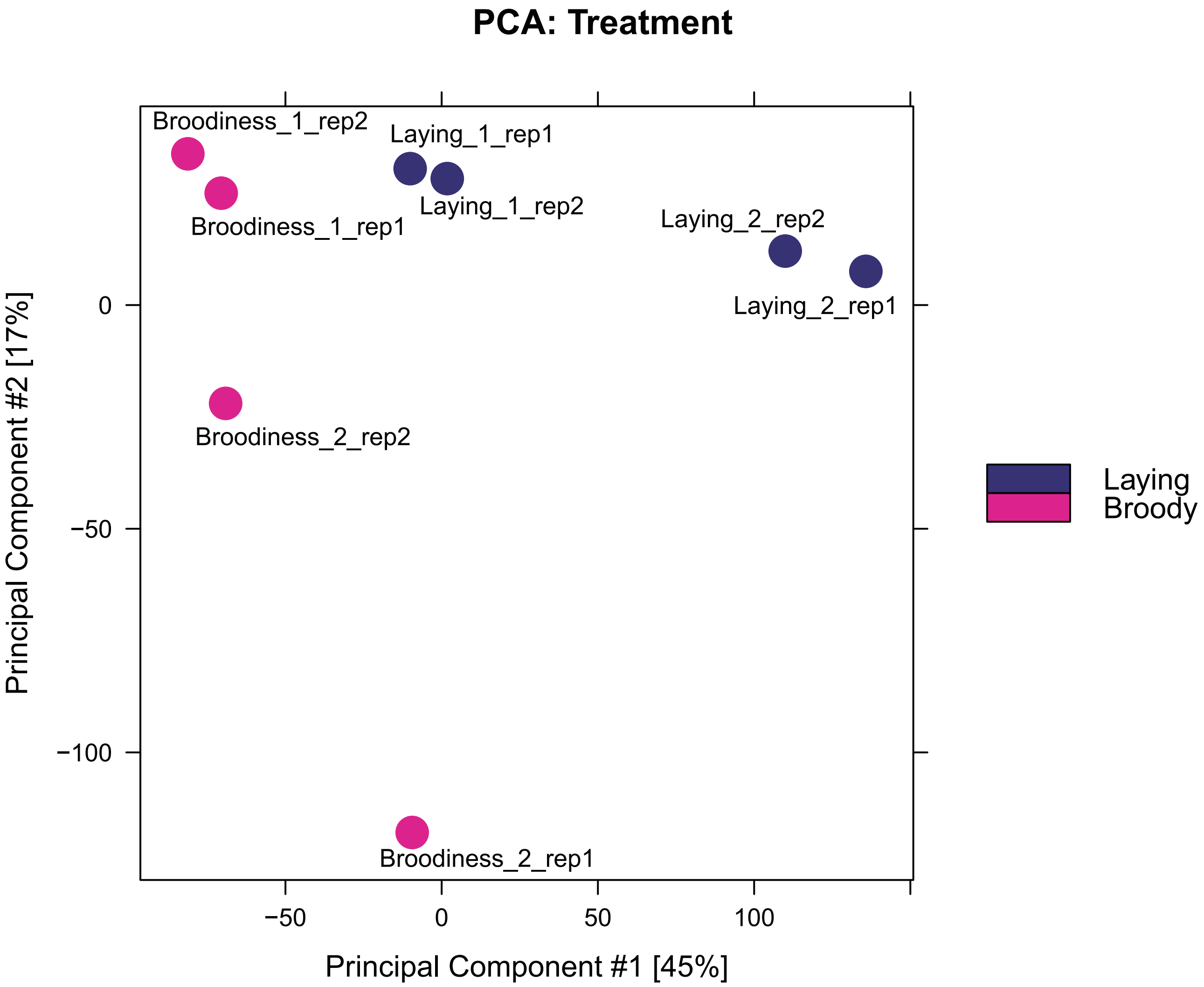

Figure S2. PCA analysis of ATAC-seq libraries.

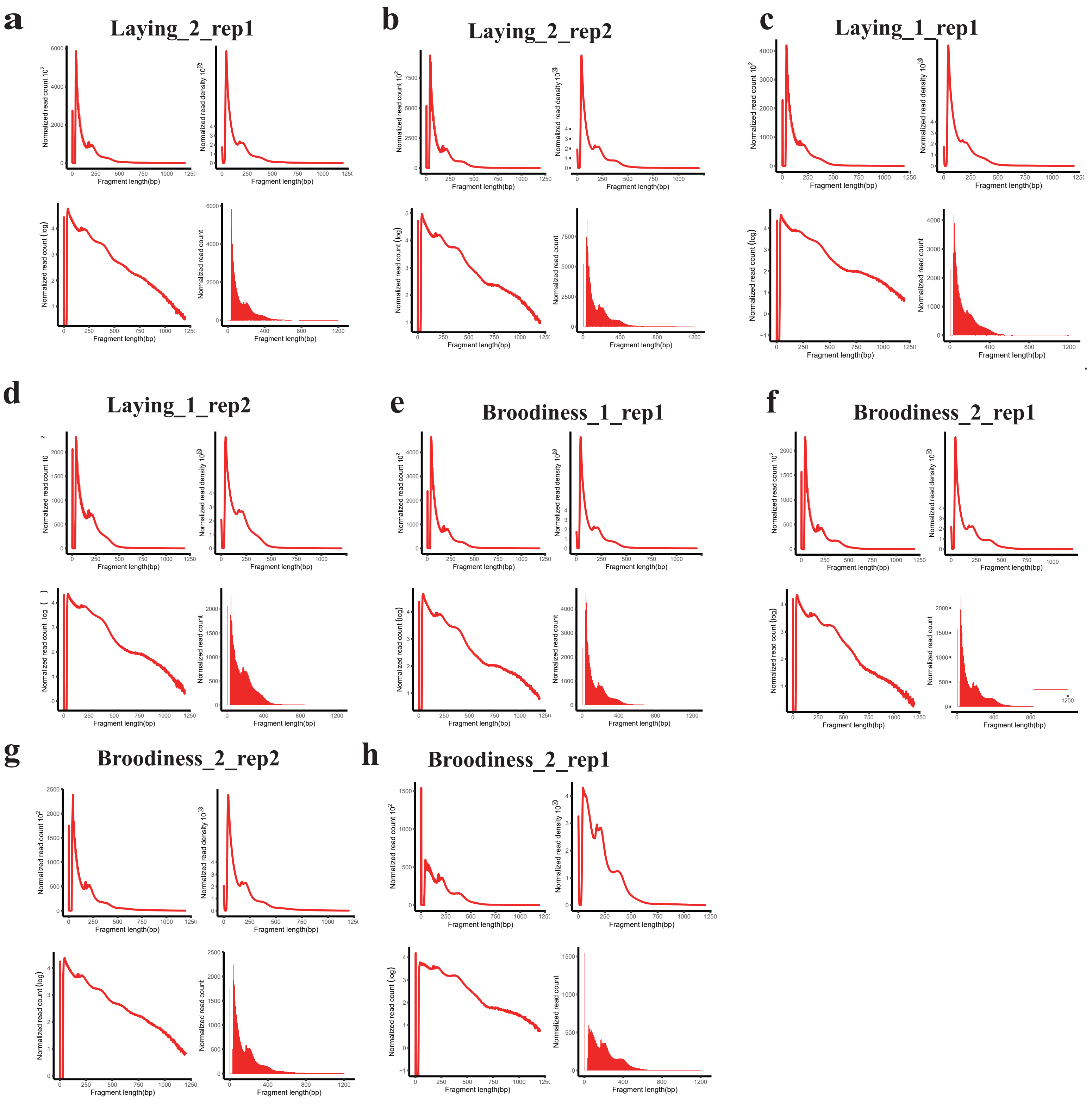

Figure S3. Insert fragment statistics of eight ATAC-seq sequencing libraries. The upper left panel counts the standardized reads, the upper right panel shows the smoothing of the left panel, the lower left panel logs the standardized reads, and the lower right panel shows the color filling of the standardized reads.

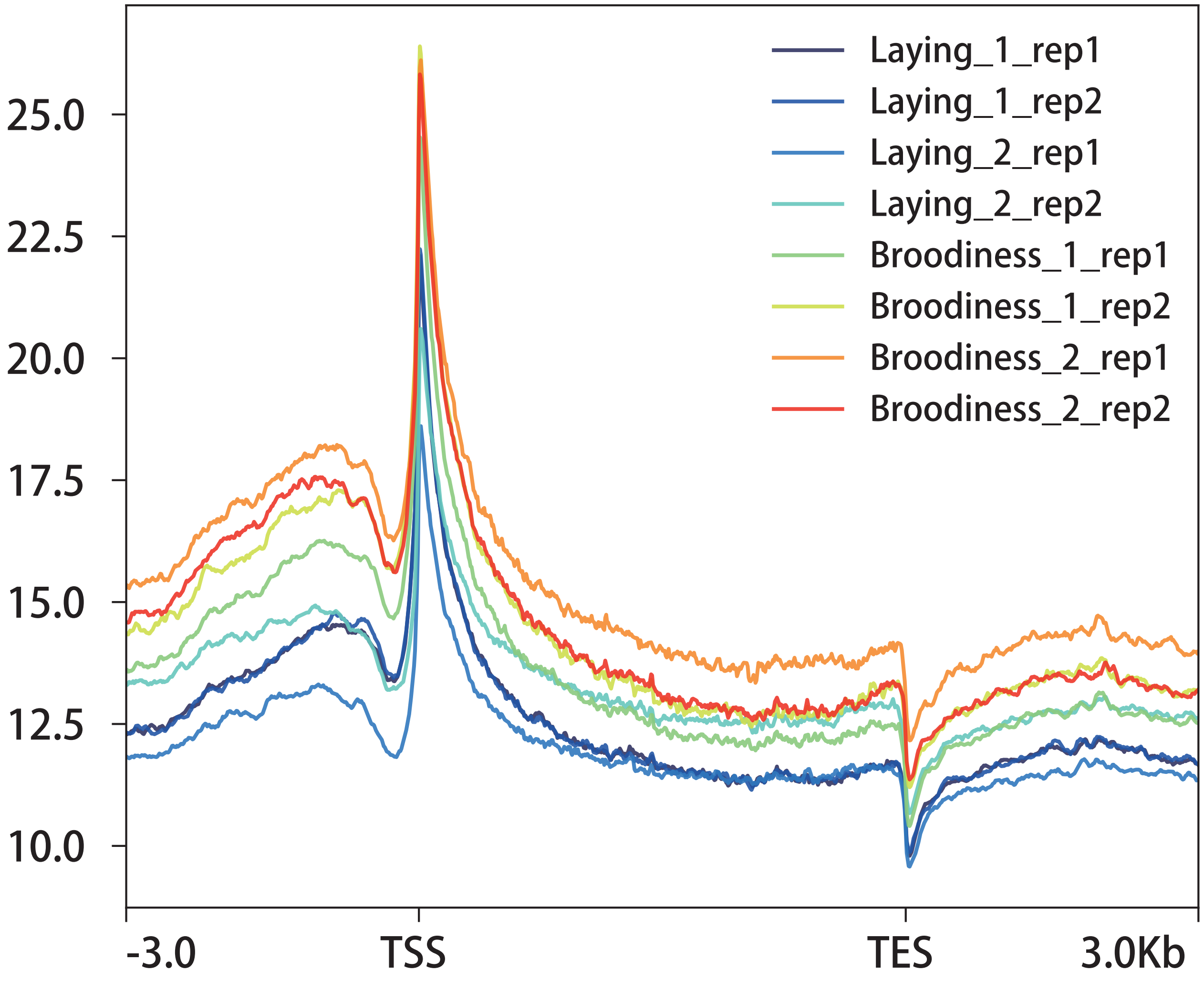

Figure S4. ATAC-seq peak signal distributions near the transcription start site (TSS) and transcription termination site (TES).The line plot shows that most of the ATAC-seq library reads clustered near the TSS region, while fewer reads were covered in the TES region.

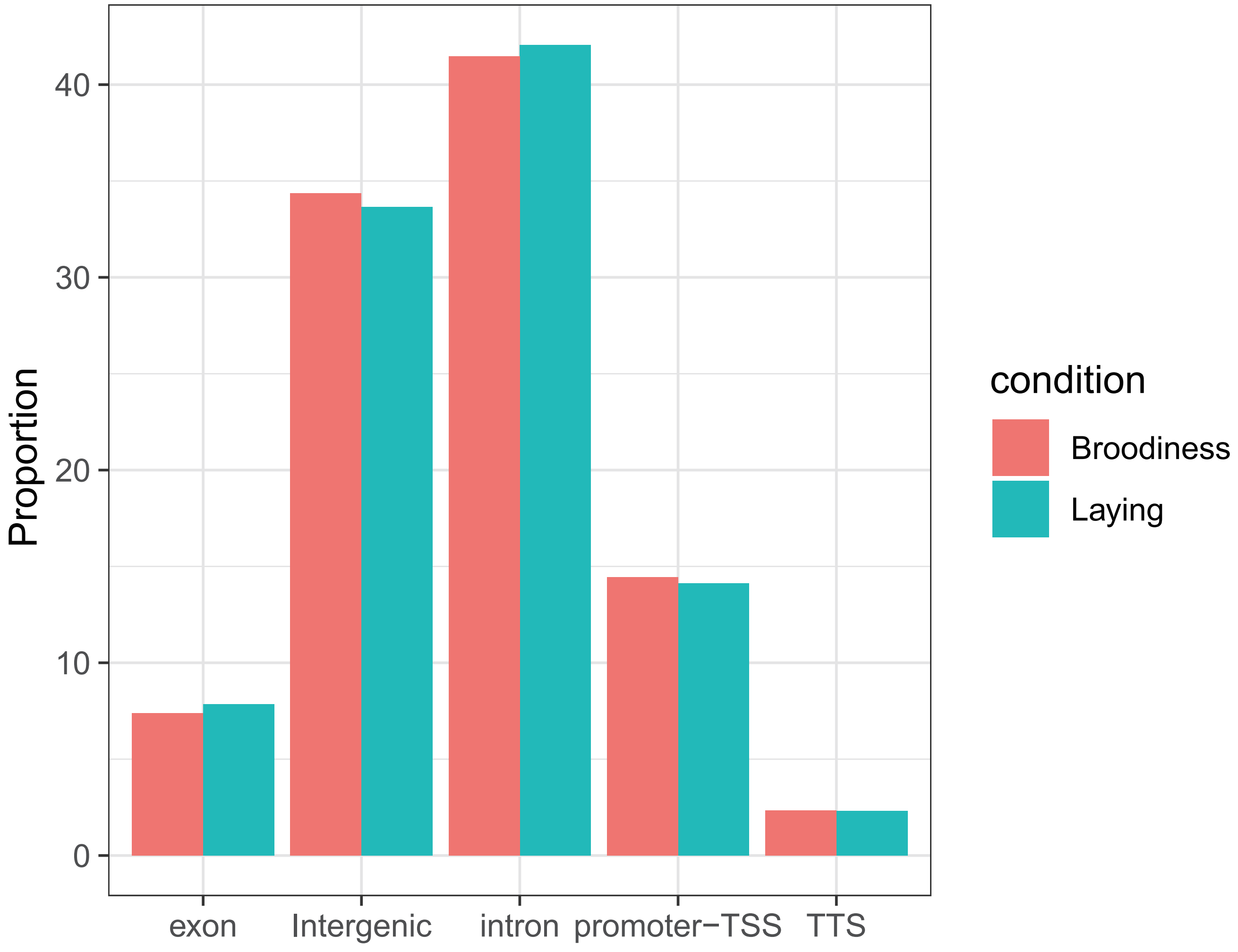

Figure S5. The Proportion of genomic locations of peak in two stage.

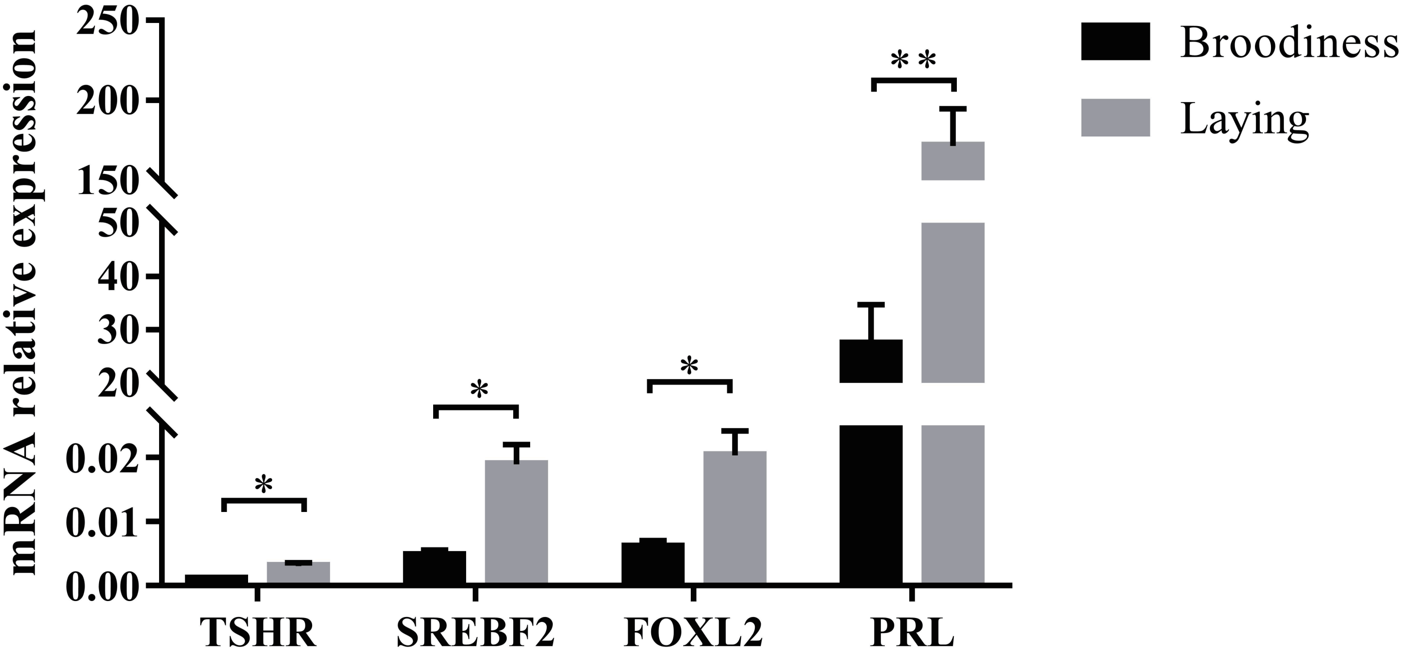

Figure S6. Verification of RNA-seq data by qRT-PCR.

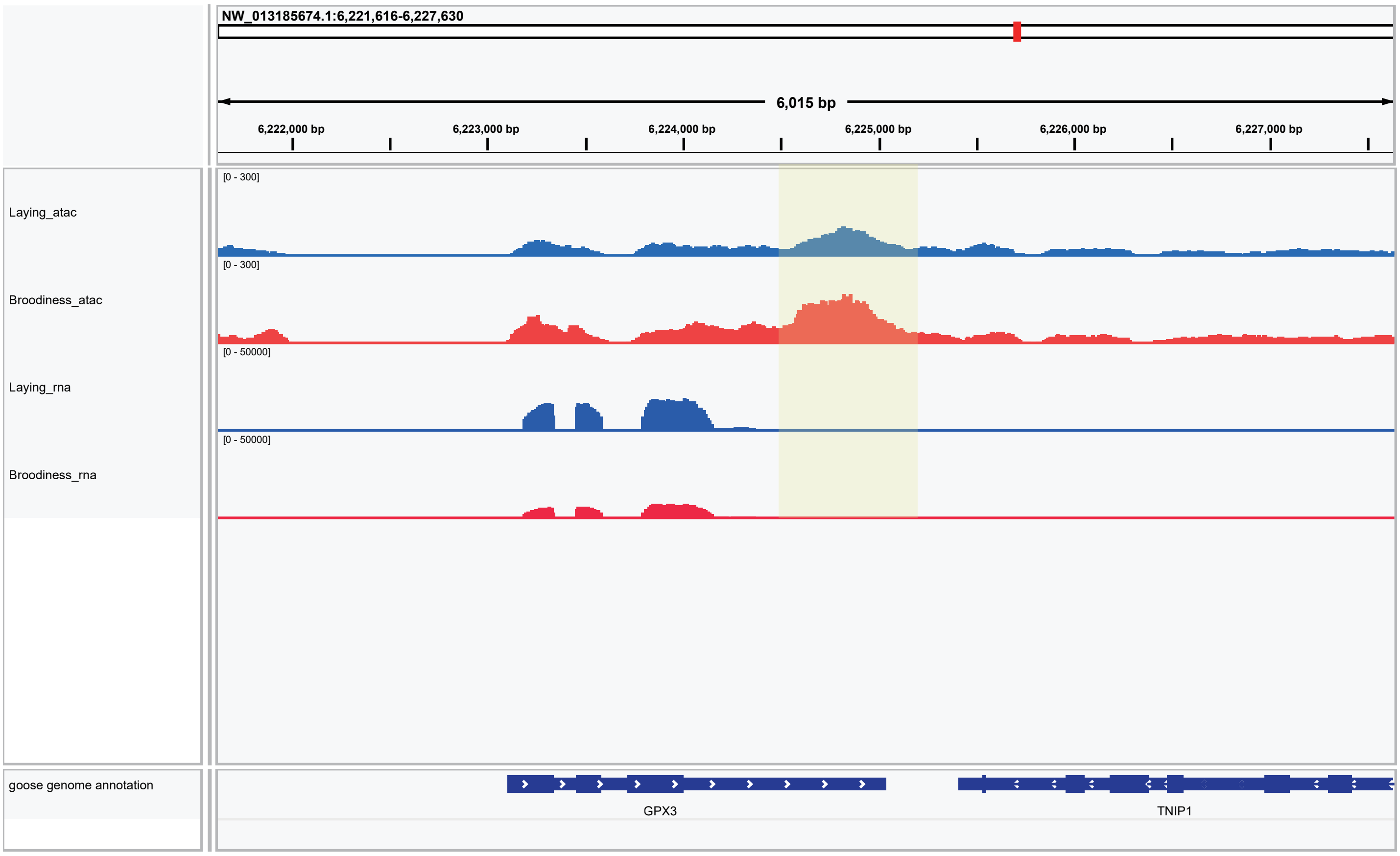

Figure S7. Comparison of open chromatin landscape and gene expression near GPX3 in laying and broodiness stages.The upper two tracks are open chromatin landscape, and the lower two tracks are gene expression. Red represents the nesting stage and blue represents the laying stage.The yellow box is DARs.

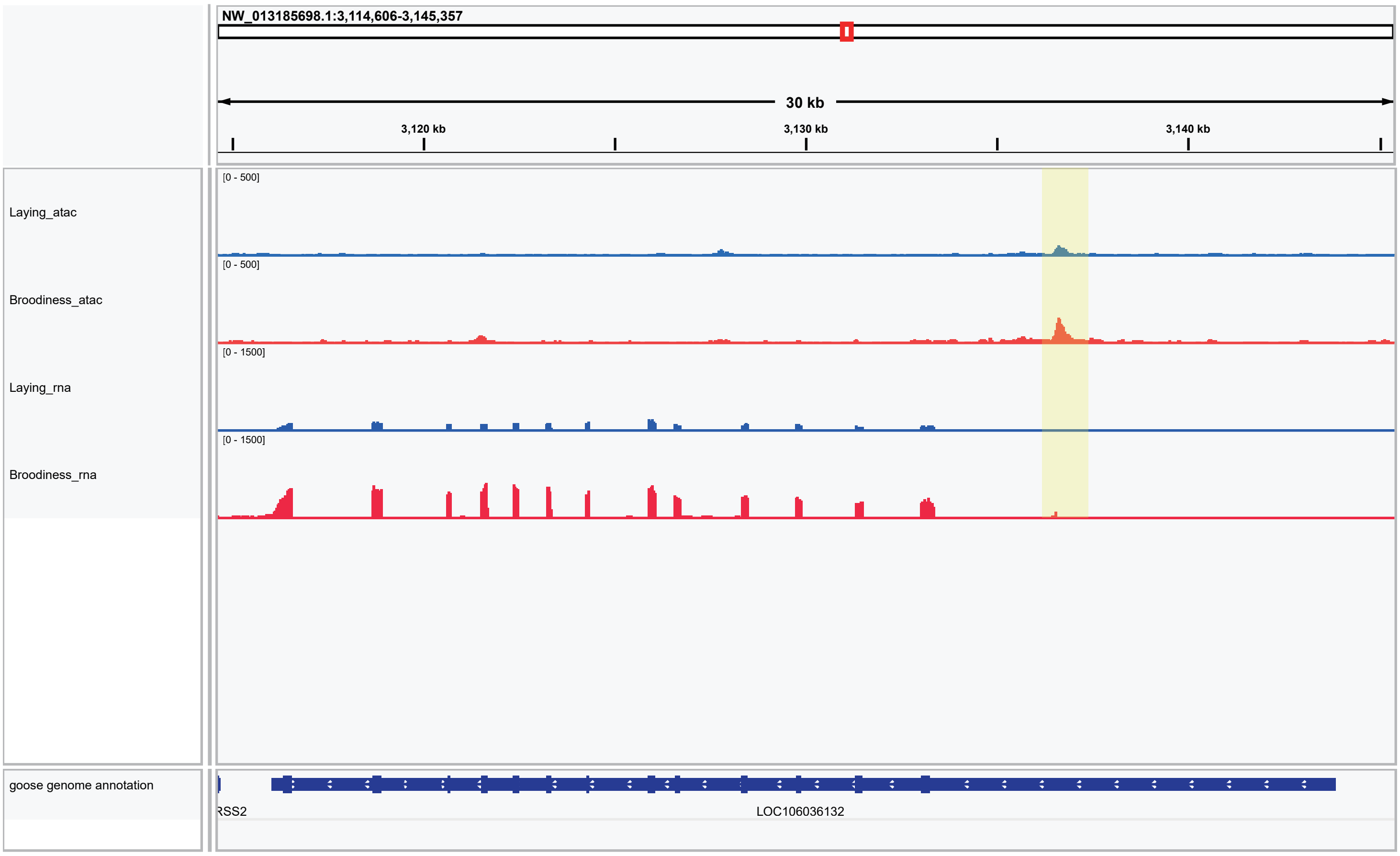

Figure S8. Comparison of open chromatin landscape and gene expression near LOC106036132 in laying and broodiness stages.The upper two tracks are open chromatin landscape, and the lower two tracks are gene expression. Red represents the nesting stage and blue represents the laying stage. The yellow box is DARs.

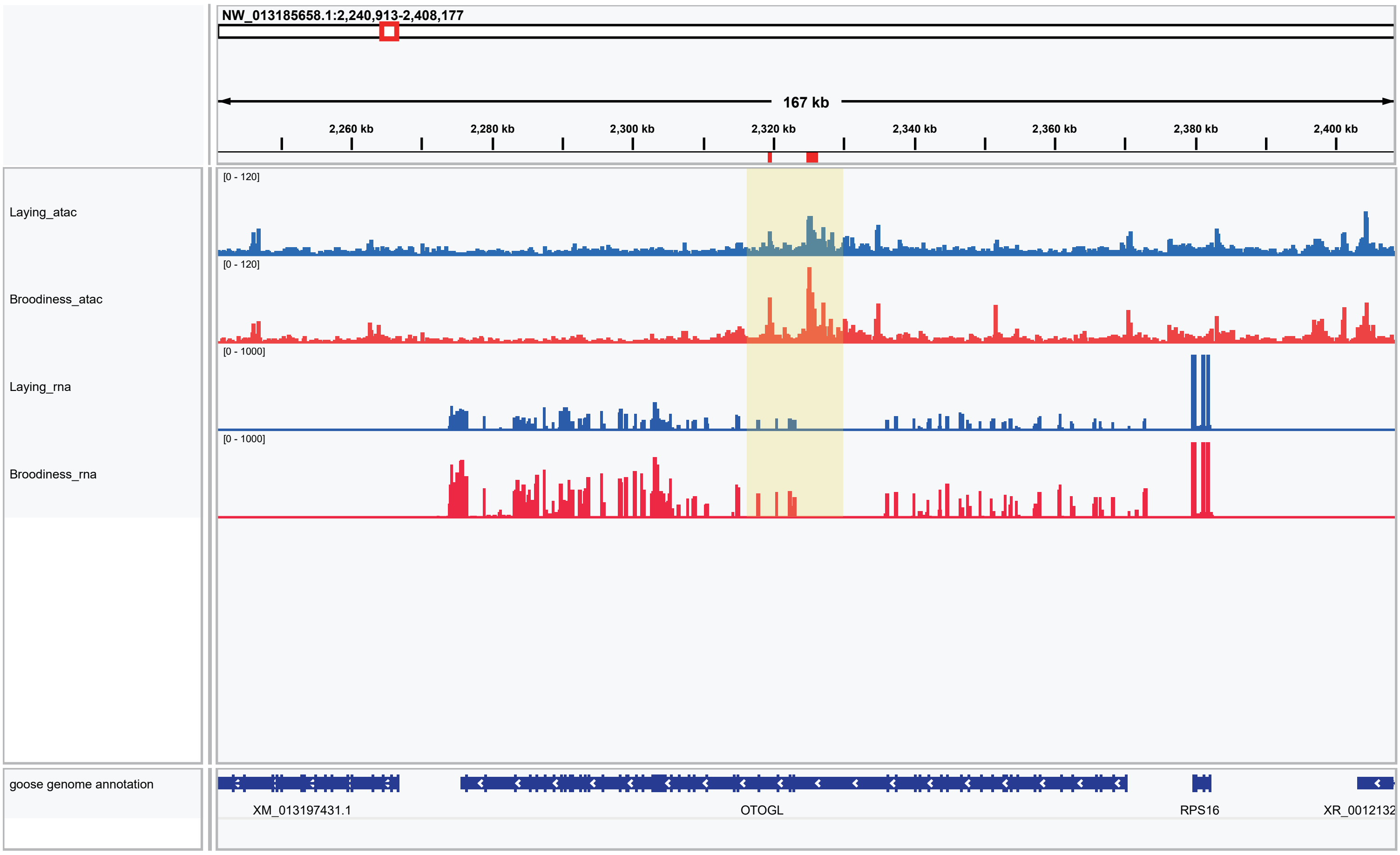

Figure S9. Comparison of open chromatin landscape and gene expression near OTOGL in laying and broodiness stages.The upper two tracks are open chromatin landscape, and the lower two tracks are gene expression. Red represents the nesting stage and blue represents the laying stage. The yellow box is DARs.

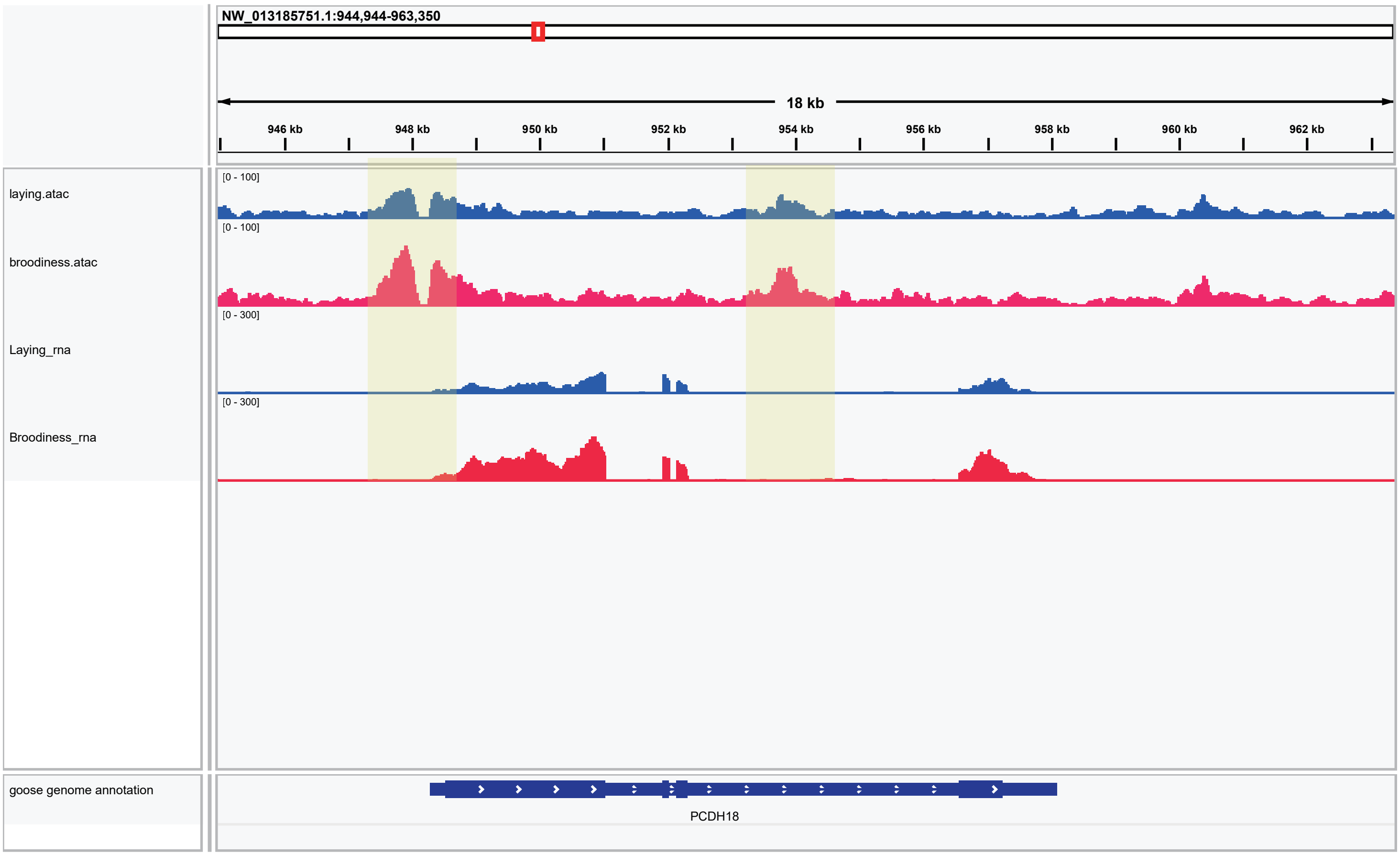

Figure S10. Comparison of open chromatin landscape and gene expression near PCDH18 in laying and broodiness stages.The upper two tracks are open chromatin landscape, and the lower two tracks are gene expression. Red represents the nesting stage and blue represents the laying stage. The yellow box is DARs.

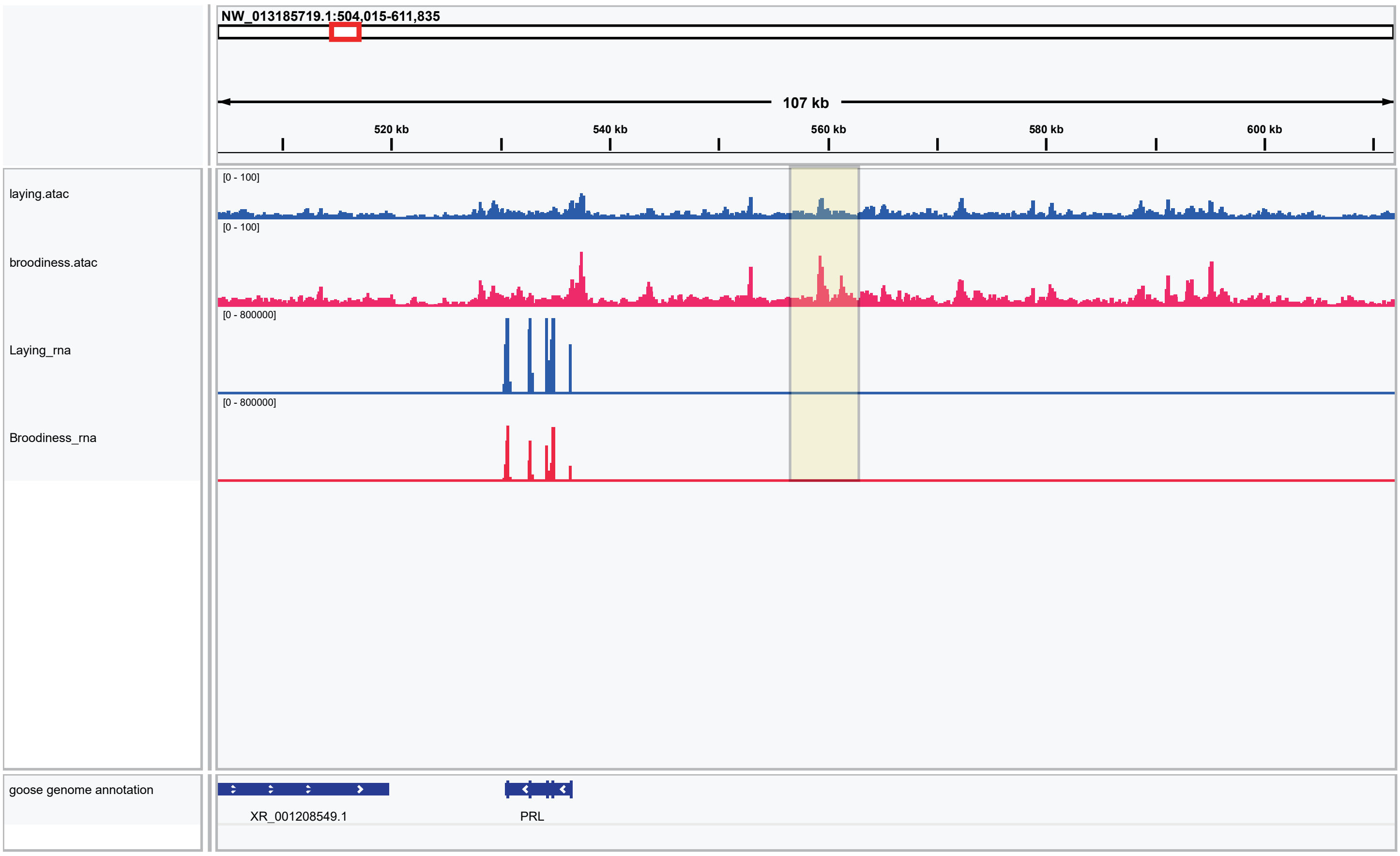

Figure S11. Comparison of open chromatin landscape and gene expression near PRL in laying and broodiness stages.The upper two tracks are open chromatin landscape, and the lower two tracks are gene expression. Red represents the nesting stage and blue represents the laying stage. The yellow box is DARs.
